# Barriers and facilitators of health among older adult immigrants in the United States: an integrative review of 20 years of literature

**DOI:** 10.1186/s12889-022-13042-x

**Published:** 2022-04-14

**Authors:** Maren M. Hawkins, Daniel D. Holliday, Lance S. Weinhardt, Paul Florsheim, Emmanuel Ngui, Tala AbuZahra

**Affiliations:** 1 University of Wisconsin-Milwaukee Joseph J. Zilber School of Public Health, 1240 N 10th St, Milwaukee, WI 53205 USA; 2 University of Wisconsin-Milwaukee College of Nursing, 1921 E. Hartford Avenue Milwaukee, Milwaukee, WI 53211 USA

**Keywords:** Older adult health, Immigrant health, Integrative review

## Abstract

**Background:**

There are over seven million older adult immigrants in the United States, and that number is expected to increase. Older adult immigrants in the United States have unique factors that influence their health.

**Methods:**

In this integrative review, we systematically review 20 years of peer-reviewed literature on the barriers (i.e. isolation, lack of English Language Proficiency, low health literacy, lack of SES resources, discrimination) and facilitators (i.e. English Language Proficiency and maintaining ones native language, social support, culturally sensitive providers, healthcare access) of health among older adult immigrants in the United States.

**Results:**

We found differing uses of the term ‘older adult’, emphasis on the lack of homogeneity among older adult immigrants, social support and isolation as significant barriers and facilitators of older adult immigrant health, and inconsistencies in uses and definitions of acculturation. We also examined relevant theories in the literature. Based on the literature review, focusing on Acculturation Theory, Social Cognitive Theory, and Successful Aging Theory, combining these three theories with findings from the literature to create the Older Adult Immigrant Adapted Model for Health Promotion.

**Conclusions:**

Public health strives to promote health and prevent adverse health outcomes. Our integrative review not only systematically and thoroughly explicates 20 years of literature, but the Older Adult Immigrant Adapted Model for Health Promotion, provides guidance for future research and interventions.

## Background

In 2018, there were 52.5 million older adults in the United States (US) [1]. Additionally, of the 44.8 million immigrants in the United States (US) [2], 7.3 million (13.9%) were older adult immigrants, meaning they were not born in the US or its territories [1]. By 2060, the US’s older adult immigrant population is anticipated to increase to 22 million [1]. In addition to the general challenges associated with aging, older adult immigrants in the US contend with unique factors impacting their health [3].

Hence, in this integrative review, we examine the factors, both barriers and facilitators, influencing older adult immigrant health in the US. We followed the Preferred Reporting Items for Systematic Reviews and Meta-Analyses (PRISMA) guidelines [4], and Torraco’s [5], and Whittemore and Knalf’s [6], recommendations for writing integrative reviews. In summary, an integrative review, “reviews, critiques, and synthesizes representative literature on a topic in an integrated way such that new frameworks and perspectives on the topic are generated” [5]. Specifically, integrative reviews contribute to, “theory development” [6] and are applicable to research, “policy and practice” [6]. Integrative reviews differ from traditional literature reviews in their generation of additional frameworks, theories, and applications.

As no previous integrative review has been done on the health of older adult immigrants in the US, this is a topic that benefits from “holistic conceptualization and synthesis” [5] to explicate innovative approaches, and to inform public health research. Furthermore, there is need for synthesis from health equity and social justice perspective. Braveman and Gruskin write, “equity means social justice” [7], and equity cannot be achieved unless there is an absence of “systematic disparities in health” [7]. As this review will demonstrate, older adult immigrants’ content with factors that drive systematic disparities in their health, thereby inhibiting health equity. We further position the importance of health equity from a public health and human rights perspective.

Thus, in this review, we will: (1) Provide background on immigrants generally and older adult immigrants in the US and situate this topic within a public health and human rights lens. (2) Explicate our methods for the literature search. (3) Review the existing body of literature on older adult immigrant health in the US. In the literature review, we synthesize main themes, and the most common health barriers and health facilitating factors among this group. We also note the main theories used and the immigrant groups of focus. (4) Discuss Acculturation Theory, Social Cognitive Theory, and Successful Aging Theory, and illustrate the benefits of integrating aspects of these theories to create the unified conceptual model, the Older Adult Immigrant Adapted Model for Health Promotion (OAHM). Finally, (5) We will provide recommendations for future research.

In 2018, 13.7% (44.8 million) of the US population were immigrants [2], and of that 44.8 million, 7.3 million were older adults [1], the largest number of immigrants in any country in the world [2]. Among older adult immigrants, meaning those over 65 years of age according to the American Community Survey [1], 58.2% identified themselves as female, and 41.8% identified themselves as male [1]. The top ten countries of origin among older adult immigrants were: Mexico, China, the Philippines, Cuba, Germany, India, Canada, the United Kingdom, Vietnam, and Italy [1]. Interestingly, older adult immigrants in the US are typically naturalized citizens [1]; 71.7% of those over the age of 65 are naturalized citizens, compared to 46.4% of those under 64 [1]. This could be due to older adult immigrants immigrating at younger ages, residing in the US longer, and hence completing the naturalization requirements [3], or because older adult immigrants who have resided in the US for several years may seek to complete the naturalization process to be eligible for benefits restricted to citizens by the 1996 Personal Responsibility and Work Opportunity Reconciliation Act (PRWORA) [3, 8]. This will be further explored in the literature review section. It should be noted that one must be a legal permanent resident in the US for at least five years before they are eligible for naturalization [9].

There are several demographic differences between older adult immigrants and older adult US-born individuals. According to the American Community Survey (2012–2016), among older adult immigrants, only 44.6% spoke English ‘very well’ [1]. Regarding education, while older adult immigrants tend to be less well educated than younger immigrants [1], immigrants overall have higher levels of post-secondary education than US-born individuals [10]. Among older adult immigrants, 27.5% have a bachelor’s degree or greater, compared to 25.2% of US-born individuals [1]. However, 31.3% of older adult immigrants had less than a high school education, compared to 15% among US-born individuals [1]. Yet, level of education varies greatly among immigrant groups. For example, only 10.6% of older adult immigrants from Latin America had a bachelor’s degree or greater, compared to 37% of older adult immigrants from Africa [1].

Furthermore, in regard to disability, the older foreign-born population was less likely to report having a disability (34.2 percent) than their US-born counterparts (36.0 percent) [1]. Thirty-six percent of US-born older adults reported a disability compared to 32.4% of older adult immigrants [1]. However, older adult immigrants had a lower prevalence of owning a home than US-born older adults [1], and older adult immigrant males had a greater prevalence of still being in the work force, even when eligible to retire, compared to US-born individuals [1]. Older adult immigrants also had a greater prevalence of living in poverty, 15.8%, compared to 8.1% among US-born older adults [1]. Finally, older adult immigrants had a higher prevalence of being uninsured, 4.9%, compared to 0.4% among US-born older adults [1]. Therefore, while older adult immigrants may report less disability, they may have fewer resources, which are important to preventing and addressing health concerns.

Yet, depicting the struggles of immigrants is insufficient, as it may unintentionally reinforce racist and isolationist sentiments. Hence, we will also note that refugees (defined on page 5) paid $21 billion in taxes in 2015 [11], and undocumented immigrants paid over $11.6 billion in taxes in 2013 [12]. Additionally, immigrants are more often entrepreneurs than US-born individuals, thereby creating jobs [11]. Immigrants, including undocumented immigrants, are also less likely to commit crimes than US-born individuals [13, 14]. Moreover, the US-born population is aging, and population growth has stagnated [15], which is leading to a workforce shortage in many states [15]. While there is debate as to whether high levels of immigration could completely solve this problem [16], it is one proposed solution as, “immigrants are more likely to be of working age, more likely to start their own business, and are more likely to work unusual hours or move for new job opportunities” [17]. However, what about older adult immigrants?

Older adult male immigrants are more likely to still be in the workforce compared to US-born older adults [10]. Although older adult immigrants are more likely to have a low income and rely on government assistance programs [10, 18], they provide unseen support. For example, some older adult immigrants migrate to support their children and grandchildren, often providing child-care [19], and this extended family can provide valuable support [20]. Additionally, as we will discuss in the literature review section, older adult immigrants have much to teach US-born individuals, such as traditional or holistic medicine [19]; and opportunities to share their knowledge is important to promoting their overall health [19]. Moreover, while the debate over deservingness in the US is hotly contested, there is also the argument that everyone, no matter from whence or where they came, deserve healthy lives. However, before continuing this review, it is necessary to clarify several definitions and terms.

### Health

In this review, we use the term health to refer to the many factors, from health insurance coverage, to the availability of public transport, which facilitate “a state of complete physical, mental and social well-being and not merely the absence of disease or infirmity” [21]. Therefore, we use the term health to reference access to healthcare and associated determinants of health. Hence, we use the term health rather broadly.

### Public health and human rights

Public health, “promotes and protects the health of people and the communities where they live, learn, work and play” [22]. Moreover, in order to address inequities and injustice in public health, we must understand the intersecting causes of these challenges. Older adult immigrants, as we will elucidate, have a nuanced relationship with the factors impacting their health. Thus, addressing and preventing negative health outcomes and promoting the factors that promote health among older adult immigrants in the US, “where they live” [22] is crucial. Health, which is impacted by access to necessities, such as social services and medical care. According to the United Nations (UN) Universal Declaration of Human Rights (UDHR):

“Everyone has the right to a standard of living adequate for the health and well-being of himself and of his family, including food, clothing, housing and medical care and necessary social services, and the right to security in the event of unemployment, sickness, disability, widow-hood, old age or other lack of livelihood in circumstances beyond his control” [23].

Employing a Human Rights perspective is vital to Public Health [24]. Thus, promoting health, and preventing and addressing health inequities, requires examining not just the individual or interpersonal factors that impact health, but structural factors such as racism and other types of marginalization common in the United States. Human Rights, woven in public health, also necessitates a view that people deserve to be healthy, and have access to the resources that facilitate health.

### Immigrant versus refugee versus migrant

According to the United Nations High Commissioner for Refugees (UNHCR), the definition of a migrant is one who elected to leave their home for a number of reasons, including family reunification, education, or work [25]. While a refugee is, “fleeing armed conflict or persecution,” [25] an immigrant is someone living in a country in which they were not born [26]. However, in regard to ‘migrant’ versus ‘refugee’, Castañeda and Holmes note that “[w]hether a person is identified as a refugee or as some other socially constructed category… depends on historical, sociocultural, political, and economic contexts” [27]. Thus, the question of choice remains debated among migrants and refugees and the definitions are not “rigid” [27–29]. This is not to say that there is not a difference between a refugee fleeing persecution and a migrant accepting a job in another country, rather that the matter is not easily defined. For example, if someone is forced to flee their home due to a natural disaster, this may be forced migration [30], but they may not meet the definition of a refugee [25]. In the literature we reviewed, refugees and immigrants (including but not limited to naturalized citizens, visa-holders, and undocumented persons) are often grouped together [29]. Hence, for the purpose of this review, the term ‘immigrant’ will be used to refer to individuals considered either immigrants or refugees.

### Older adult versus elderly

In this literature review, we found a shift in 2000–2021 from the use of the term ‘elderly’ to the term ‘older adult’. The article, “Use of the Term ‘Elderly’” [31] published in 2012, reflects this shift, arguing that the term ‘elderly’ may be considered ageist and is laden with value judgements [31]. Therefore, throughout this review, we use the term “older adult.”

## Methods

### Search methods

We conducted a systematic search of the literature following PRISMA guidelines [4]. We used the following six search engines: CINAHL, Global Health, Google Scholar, PsycINFO, PubMed, and Sociological Abstracts, and restricted our search to the years 2000–2021. We also used citation chaining to identify additional relevant articles. As we wrote this review at the beginning of 2021, the only works included from 2021 are those published between January 1st and February 7^th^ of 2021.

For our search, we used the following 12 search terms: For our search, we used the following 12 search terms:

1. “older adult immigrants” OR “older adult refugees” AND “health”,
2. “older adult immigrant” OR “elderly immigrant” OR “older adult refugee” OR “elderly refugee” AND “Health”,
3. “older adult immigrants” OR “older adult refugees” AND health concerns or health problems or health consequences,
4. “older adult immigrant” OR “elderly immigrant” OR “older adult refugee” OR “elderly refugee” AND health concerns or health problems or health consequences,
5. “older adult immigrants” OR “older adult refugees” AND health facilitators or facilitators to health promotion, and
6. “older adult immigrant” OR “elderly immigrant” OR “older adult refugee” OR “elderly refugee” AND health facilitators or facilitators to health promotion.

For PubMed we used the additional terms of:

(7) “older adult” AND “older adult immigrant” OR “older adult refugee” AND “health”,
(8) “older adult” OR “elderly” OR “older adult immigrant” OR “elderly immigrant” OR “older adult refugee” OR “elderly refugee” AND “Health”,
(9) “older adult” OR elderly” OR “older adult immigrant” OR “elderly immigrant” OR “older adult refugee” OR “elderly refugee” AND health concerns or health problems or health consequences,
(10)  “older adult” OR “elderly” “older adult immigrant” OR “elderly immigrant” OR “older adult refugee” OR “elderly refugee” AND health facilitators or facilitators to health promotion. Finally, for Google Scholar, we used the additional terms,
(11)  “older adult immigrant” OR “elderly immigrant” OR “older adult refugee” OR “elderly refugee” AND “Health” AND “United States” NOT “Canada” NOT “Israel” NOT “Europe”, and.
(12)  “older adult immigrant” OR “elderly immigrant” OR “older adult refugee” OR “elderly refugee” AND health concerns or health problems or health consequences AND “United States” NOT “Canada” NOT “Israel” NOT “Europe”, in addition to terms #1, #3, #5, and #6.

We conducted the searches with and without the inclusion of the term ‘elderly’ because there was a transition around 2010 from the use of the term ‘elderly’ to the term ‘older adult.’ We had several inclusion and exclusion criteria depicted in Table 1:

**Table 1:** Inclusion and Exclusion Criteria

Inclusion Criteria	Exclusion Criteria
• Peer-reviewed articles, including quantitative, qualitative, reviews, and conceptual articles • Published between January of 2000 and February 7th of 2021 • Specific to, or included, the United States • Focused on health, or a health facilitating factor such as social service provision • Focused on a health, or a barrier to health, such as lack of transportation • Focused solely on, or included, older adults • Focused on immigrants or refugees	• Non-peer reviewed papers, such as theses and dissertations • Commentaries • Opinion pieces • Instrument validation • Educational guides (ex: exercise curriculum, or on pedagogy for teaching students about older adult immigrants.) • Not specific to older adults • Not specific to immigrants or refugees • Specific to minority US-born citizens. For example, older adult Latinos born in the U.S • If the article focused solely on the caregivers of older adult immigrants but did not include the older adult immigrants • Specific to Puerto Ricans moving from the island of Puerto Rico to the continental US • Clinical practice recommendations • Specific to return migration

### Search outcome

Our search in the six databases using 12 search terms yielded 3,381 results. We screened these results based on their title and abstract to determine what to include for full-text screening. Among those 3,381 results, there were 693 duplicates, and based on our inclusion and exclusion criteria, we screened 352 full-text articles. In total, 145 articles met our inclusion criteria.

**Fig. 1:**
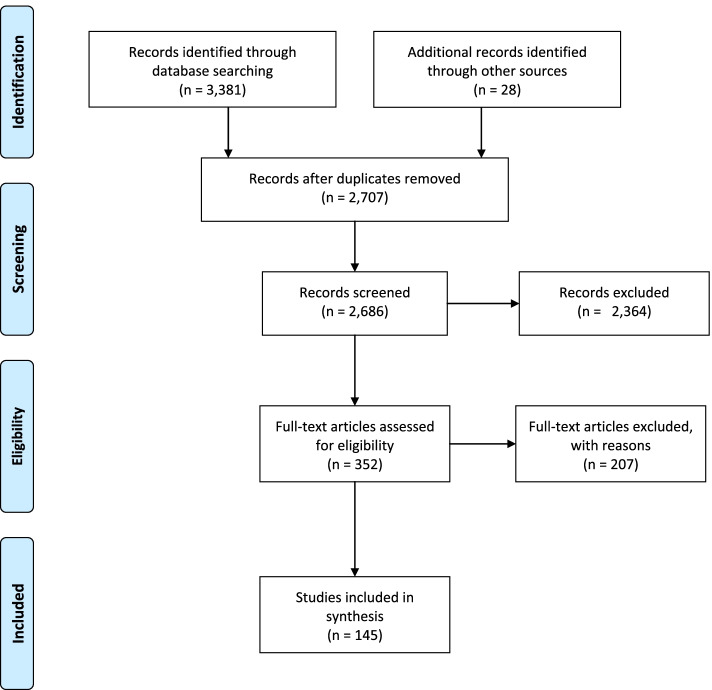
PRISMA Diagram created by research team

For the 145 articles included, we created an excel spreadsheet, which includes key information about the articles included, specifically: The citation, year of publication, type of article, population of focus, health topic of focus, number of participants, age of participants, reported sex or gender of participants, theory, framework, or model used, methods, and whether the study was an intervention. We created a PRISMA flow-chart (Fig. 1) to document our search.

## Results

Of the 145 articles included, 85 were quantitative, 44 were qualitative, three were mixed methods, eight were reviews, and five were conceptual.

### Populations of Focus

There were 32 different groups of focus in the articles. Overall, the top three immigrant groups of focus, after older adult immigrants generally, were those from the People’s Republic of China, the Republic of Korea (South Korea), and those from the Former Soviet Union (FSU). Other groups discussed in the articles included, Hispanic/Latinx, Asian, and African people generally, and Kurdish people. As well as those from Mexico, the Dominican Republic, Colombia, Guatemala, Cuba, Taiwan, the Philippines, Vietnam, Japan, Bhutan, Afghanistan, India, Iran, Somali, Nepal, Myanmar (Burma), Liberia, Burundi, and Liberia.

### Health topics of focus

In the studies, more than 50 health conditions and factors impacting health were examined. Overall, access to and use of healthcare services; health insurance coverage; mental health; including depression; activity participation; and English language proficiency, were the most common health inhibiting and facilitating topics discussed. In some studies, a single health outcome, such as diabetes, was discussed. Other articles instead examined the relationships between multiple factors, such as examining the relationship between acculturation, health beliefs, and health care use [32]. Social support and acculturation were the two most common factors examined in relation to health and health conditions. Both social support and acculturation will be discussed in the subsequent findings section.

### Theories and frameworks of focus

In five of the 145 studies, authors developed their own conceptual framework. Additionally, in 47 studies the authors explicitly discussed and drew upon a specific theory, framework, or model to inform the study. Nearly every theory, framework, or model, explicitly used by the authors was different. Only four frameworks were used in more than one study. An Ecological framework was used in five studies, and only two of those studies used the same one, those frameworks were: An Ecological Framework [19, 33], an Ecological Model [34], Ecological Systems theory [35], and an Ecological Theory of Aging [36]. A Life Course Perspective was used in five studies [37–41]. Finally, three different studies used Acculturative frameworks [42–44] and two studies used Kleinman’s Exploratory Model [45, 46]. Notably, acculturation was commonly mentioned, yet seldom used as an explicit framework. This is further discussed in the findings section.

Drawing from the 145 articles, we created Table 2 to show the barriers and facilitators of health and well-being for older adult immigrants in the US. These barriers and facilitators are organized using the Social-Ecological Model (SEM) [47]. Three of the barriers and facilitators appear in more than one level. Following this table, we discuss the main themes, which were: (1) More females than males, (2) differing uses of the term ‘older adult’, (3) older adult immigrants are not homogeneous, (4) isolation and social support, (5) age-at-migration matters, (6) acculturation inconsistencies, and (7) learning from older adult immigrants.

**Table 2:** Barriers and Facilitators of Health and Well-Being among Older Adult Immigrants in the United States

	**Barriers**	**Facilitators**
Individual	- Lack of English language proficiency - Refugee status (as a proxy for trauma) - Loss of independence - Low health literacy - Loss of a spouse - Lack of SES resources - Loss of independence - Dementia/memory loss - Poor physical health - Depression - Chronic pain - Chronic conditions - Sleep issues/Insomnia	- Owning a personal vehicle/ability to drive **-** English language proficiency **-** Formal education in the U.S - Resilience - Positive emotions (ex: optimism, positive affect) and psychological well-being - Maintaining one’s native language and traditions - Traditional medicine/Holistic approaches to health - Life satisfaction - Proper nutrition - Leisure time - Sexual health knowledge
Interpersonal	- Isolation - Social exclusion - Discrimination based on racial/ethnic/gender identity - Loss of previous social status - Financial abuse - Older adult abuse - Healthcare providers misinterpreting immigrants health service expectations and feeling unprepared to work with immigrants - Expectational role friction	**-** Social support - Culturally sensitive providers/culturally appropriate care - Receiving professional care -Provider and treatment trust - Opportunities for them to share their life experiences and knowledge with others, such as teaching cuisine/food - Liaisons, “helpers” from the immigrant community to aid other immigrants [48] - Social capital - Trust
Organizational	- Discrimination based on racial/ethnic identity - Limited access to support services - Lack of preventative care - High cost of medical care/financial concerns - Issues accessing medical care	- Healthcare access - Translated materials—that are culturally relevant - Provision of culturally relevant information - Traditional medicine/Holistic approaches to health -Health education - Participation in activities (ex: social, cognitive, spiritual/religious, physical) - Culturally meaningful activities - Resource access (in addition to healthcare) - Group excursions, or “field trips”^18^ - Home health care services
Community	- Lack of available, and inaccessible, transportation -The built environment – Lack of safety and walkability - Poverty - Discrimination based on racial/ethnic identity	- Neighborhood cohesiveness - The built environment - Available public transport - Healthcare access - Availability of care - Community trust
Structural	- Discrimination based on racial/ethnic/gender identity - The 1996 Personal Responsibility and Work Opportunity Reconciliation Act - Lack of citizenship/temporary status - Lack of insurance	**-** Citizenship - Health insurance

### More females than males

In 2018, 58.2% of older adult immigrants in the US identified as female, and 41.8% identified as male [1]. However, in the 145 studies, in only 13 studies were there more females than males.

Differing uses of the term ‘Older Adult’.

There were several different ages used to encompass ‘older adults’ in the literature. According to the US Census Bureau, the American Community Survey, and HealthyPeople.gov, an older adult is one 65 and older [1, 49]. Yet, according to a 2012 report from the Centers for Disease Control and Prevention (CDC), an older adult is someone age 60 or older [50]. Differing definitions and age limits in were present in the studies reviewed. While many articles used 65 years or older as their inclusion criteria, others selected a lower age, still categorizing the age as the threshold for ‘older adult.’ For example, Cofie et al. [51], Zhang and Zhan [38], Nandan [48], and Gautam et al. [52], included those 50 and older; while Guo et al. [53], Lee and Eaton [54], Sohng et al. [55], and Aroian and Vander [56], included those 60 and older. This creates concerns when comparing studies, especially when assessing health insurance, as one needs to be at least 65 to qualify for Medicare unless qualifying via disability status or in few other conditions [57]. It also elicits a more conceptual question, who is an older adult?

### Older adult immigrants are not homogeneous

The literature emphasized that older adult immigrants are not a homogeneous group, and interventions cannot treat all older adult immigrants the same. Nandan [48] noted in their qualitative study with older adult immigrants from India that, not only are immigrants from the continent of Asia not homogeneous, but Indian immigrants are unique in that many already speak English before arriving in the US [48]. However, tensions in values still exist [48]; and many participants in Nandan’s study felt that their spiritual needs were not met [48]. Similarly, Kang et al. [58], in their qualitative study with older adult Korean and Chinese immigrants, found that for the two groups, different factors impacted their healthcare utilization. For older adult Chinese immigrants, gender and length of stay in the US impacted healthcare use, while for older adult Korean immigrants, marital status was a stronger predictor of healthcare use [58]. However, for both groups, lack of English language proficiency, lack of health insurance, cultural tensions, and the presence of depressive symptoms all negatively impacted healthcare service use [58]. Additionally, Mui et al. [39], in their quantitative study with older adult Korean immigrants, Chinese-immigrants, and US-born individuals, found that more older adult Korean immigrants reported that religiosity was a coping mechanism than older adult Chinese immigrants [39]. Yet, similarly to Kang et al., Mui et al. found that for both immigrant groups poor English proficiency was associated with worse health outcomes [39].

Notably, the emphasis on the lack of homogeneity in immigrant groups reflected a tension in the literature regarding an emic versus etic approach [59], and a broader debate about the generalizability of findings. Essentially, there was tension between the studies which grouped older adult immigrants together (30 studies grouped older adult immigrants together), and those focusing on a specific immigrant group. Furthermore, there was also tension in the studies that focused on a single specific group of older adult immigrants. This was the case with many studies focusing on older adult Chinese immigrants. Some studies specified that Chinese immigrants included those from China, Taiwan, or Hong Kong, or that Chinese immigrants included Mandarin, Fujianhua, and Cantonese speakers [60, 61]. Some studies differentiated immigrants from mainland China and those from Taiwan [62]. Older adult immigrants are not homogeneous, and future studies should be clear about how they define groups. Nonetheless, these emic and etic studies provide valuable information on factors impacting health among older adult immigrants in the US, especially when the same findings (listed in Table 2) were echoed in small qualitative studies and studies using large nationally representative samples.

### Isolation and social support

Isolation and social support were two of the most common health-influencing factors discussed in the literature. Social support is especially interesting because, while over 20 studies found that social support was an important factor, only 10 studies focused on social support in their article conceptualization. This means that even when researchers did not set out to examine the importance of social support, it continued to emerge as a crucial factor. Social support is a broad concept and is generally conceptualized as having friends or family on whom one can rely [63]. Additionally, in the literature, social support also included social networks and social relationships. One study by Rhee [64]. found that social support, or a lack thereof, “was the strongest predictor of depression” [64] for older adult Korean immigrants, moreso than somatization and acculturative stress. Cummings et al. [65], found the same finding among older adult Kurdish refugees, that a lack of social support was a significant predictor of depression. A key caveat however, found by Liu et al. [66], was that social support needed to be healthy, and that negative social support could be detrimental to one’s mental health.

Isolation lies opposite of social support. Isolation, meaning to be separate from others [67], was linked to English language proficiency. Tran et al. [68], found that isolation combined with a lack of English language skills resulted in older adult immigrants from the FSU being unable to complete tasks or participate in “social activities” [68]. Furthermore, Serafica [69], identified isolation as contributing to emotional distress among older adult Filipino immigrants. In another study by, Serafica [70], they found that improved English language skills and social relationships were protective against isolation among older adult Filipino immigrants. However, while Wang [71], found that isolation was worsened after the death of a spouse among older adult Chinese immigrants, they importantly noted that isolation was not necessarily alleviated by residing with others, even with family members. Zhang and Zhan [38], found in their study with older adult Chinese immigrants that many described feeling like they were in a “prison” [38] even though they lived with family members. This was due to a lack of English language proficiency, few friends, shifting roles with their children, and a “lack of belonging” [38]. This suggests that studies should include robust measures of isolation, not simply physical isolation.

### Age-at-migration matters

Age-at-migration, or the age at which one migrates, was related to overall health. Gubernskaya wrote that age-at-migration was a proxy measure that captures, “the degree of health selectivity upon arrival…and the length of exposure to the environmental conditions in countries of origin” [18]. In essence, if one immigrates at an older age, they were exposed to potentially adverse conditions in their country-of-origin for a longer period of time. Yet, if one immigrates at an older age, this may also reflect a degree of good health, as the immigration process is neither easy nor stress-free. In their 2015 study, Gubernskaya found that Hispanics who immigrated after age 50, had a more rapid decline in their health compared to non-Hispanic immigrants and US-born older adults [18]. Yet, interestingly, Hispanics who immigrated at age 18 still experienced sharp declines in their health after 50, which Gubernskaya partly explains as a consequence of discrimination, poor, hazardous, and low-paying working conditions, and “underinsurance, and limited access to non-emergency health care” [18] in the US.

In a 2013 study, Gubernskaya also explored age-at-migration within the context of naturalization. They use naturalization in two ways, first as a proxy for social integration, and second that naturalization is “a key indicator of social and political inclusion” [3]. They found that among naturalized citizens, those who immigrated after age 50 had worse health than those who immigrated as children or young adults [3]. They also posited that older adult immigrants who naturalize may do so because of old age and accompanying health issues, in a phenomenon called negative health selection [3]. Many benefits, such as TANF and Medicaid, were restricted to citizens by the 1996 PRWORA [3, 8], hence older adult immigrants may seek to complete the naturalization process to be eligible for benefits restricted to citizens. Hence, Gubernskaya posits that negative health selection is why naturalized citizen older adult immigrants may have greater odds of functional limitations than non-citizen older adult immigrants [3]. They also make the important point than immigrants who migrate at older ages have very limited opportunities for building resources, specifically socioeconomic resources, such as savings or retirement funds [3]. Other authors found similar and differing results.

Alemi et al. [72], in their study with older adult Afghan refugees, found that migrating at an older age was associated with worse health, specifically, psychological distress. Similarly, Nkimbeng et al. [73], in their review of correlates of disability among older adult immigrants, also found that migrating at an older age was associated with worse health. Yet, Mehta et al. [74], found different results. They found that, not only did immigrants who migrated at older ages have a longer life expectancy (+ 2.4 years) than US-born older adults, but that immigrants who migrated at older ages also had lower rates of mortality than US-born older adults [74]. Mehta et al., found the increase in life expectancy was present across all immigrant groups: those from Asia, “Central America, western/eastern Europe, and Africa.” [74] Moreover, Holmes et al. [75], examined the impact of age at migration, nativity, and length of time in the US, on mortality, specifically within older adult Hispanic immigrants, who migrated at different ages, and US-born Hispanics. They found a “mortality advantage” [75], among older adult Hispanic immigrants who migrated after age 24, compared to Hispanics who migrated before the age of 18 [75].

Furthermore, Thomson et al., similar to Gubernskaya, found that Hispanic immigrants who were citizens had higher rates of disability compared to non-Hispanic White immigrants [76]. Choi, similar to Mehta, found that older adult immigrants who migrated at older ages had better health than immigrants who migrated at younger ages and US-born older adults [77]. Yet, several factors, such as different approaches, research questions, health topics of focus, and methods of dividing immigrant groups for analysis, could drive the difference in findings between these strong studies. Overall, it seems that age-at-migration matters, although whether it is related to greater or reduced mortality warrants further exploration.

### Acculturation inconsistencies

Acculturation was the most discussed concept in the literature, but not the most explicitly applied framework. Nineteen studies examined acculturation, either as the outcome of interest or in relation to the outcome of interest, such as the impact of acculturation on health service use [78]. Additional studies heavily emphasized it in the literature review section. Yet, only three studies named an acculturative model or acculturative framework as their guiding framework. Those three studies discussed three frameworks, which were: The Bidimensional Acculturation Model [42, 79, 80], the Acculturation Model [44, 81], and the Ecological Acculturative Framework (EAF) [43, 82]. Berry [79, 80, 83–86], was the most commonly cited author on acculturation.

Moreover, the measures of acculturation differed in nearly every study. For example, some studies simply measured acculturation as length of stay in the US and English Language Proficiency (ELP) [65, 87], while others, such as Mao et al. [60], used robust measures of acculturation. Under the umbrella of acculturation, Mao et al. examined Behavioral Acculturation, Cognitive Acculturation, and Identificational Acculturation [60]. Behavioral Acculturation included daily habits, language concerns, and dependence on interethnic networks [60]. Cognitive Acculturation included, “self-contentment, fatalism, and collectivism” [60], and “individualism and independence” [60]. Finally, Identificational Acculturation included maintenance of ethnic identity [60]. Additionally, in the studies that measured acculturation, they found that acculturative stress, the stress caused by this cultural, linguistic, economic, and role re-negotiation, can lead to adverse health outcomes. Rhee found that acculturative stress was a “significant risk factor for depression” [64] among older adult Korean immigrants not residing in ethnic enclaves [64]. Similarly, Serafica found that older adult Filipino immigrants who had high levels of acculturative stress, “reported lower physical and mental health scores” [70]. However, it is difficult to make claims on the impact of acculturation and acculturative stress due to the differences in measurement.

The inconsistencies in the measurement of the concept acculturation echoes the writings of others. For example, Gubernskaya wrote that, “the concept of acculturation is not well defined” [18, 88, 89] and it is difficult to differentiate between the impact of length of stay in the US and normal “age related health declines” [18]. For these reasons, in Table 2, we never explicitly list ‘acculturation’, rather we list the specific elements measured in the studies, such as ELP. Due to this inconsistency in this concept, we will clearly define acculturation in the theoretical section, and seek to avoid common concerns with acculturation, such as inadvertent stereotyping [89].

### Learning from older adult immigrants

Finally, an important point made in the articles was what others must learn from older adult immigrants. Not only do older adult immigrants provide a wealth of knowledge on language, culture, and history, preserving that which is important to their health [19, 32, 90–95], but they also have different methods of approaching health. For example, Martin found that among older adult Iranian immigrants, health not only included physical, but also spiritual and emotional health [95]. Martin discussed the tensions caused by the biomedical model in US healthcare [95], and overall, they argue that a holistic approach to health, which recognizes that spiritual and mental health can impact physical health, and visa-versa, is important for promoting health.

In addition to holistic health, herbal medicine, or traditional medicine, when used safely with provider knowledge [93, 96], is not only important for providing culturally congruent care but are also beneficial for non-immigrant groups. For example, acupuncture has been found to aid in cancer pain management [97]. Chinese herbal medicine has also been found to be beneficial in the treatment of cardiovascular disease [98]. Additionally, in the treatment of ulcerative colitis, some herbal medicines are quite safe [96]. Van Son found that older adult immigrants from the FSU preferred herbal remedies, but emphasized the safety risks involved in mixing medications without support from a physician [93]. Ivanov and Buck found in their study with older adult immigrants from the FSU that participants also valued herbal medicine, but also spoke highly about medical care in the US when they had clear communication with providers [94]. Furthermore, in a study with older adult Korean immigrants, Kim also found that participants preferred a mix of traditional Korean medicine (hanbang) and Western medicine [92]. In their study with older adult Chinese immigrants about traditional Chinese medicine (TCM) (中医), Dong et al. found that, “TCM users are unlikely to disclose use to their physicians” and “Chinese older adults who report better quality of life also report use of TCM, suggesting the cultural relevance of TCM” [91]. These findings demonstrate a need for providers to have honest conversations with their patients about traditional/herbal medicine, and the need for greater integration of traditional medicine approaches in medicine and public health practices in the United States.

### A unified conceptual model

As demonstrated in the literature review section, many factors influence older adult immigrant health, and there is a lack of commonly used models for the health of older adult immigrants. For the unified conceptual model, we combine aspects of Acculturation Theory (AT), specifically using Ward and Geeraet’s process model of acculturation [99], Social Cognitive Theory (SCT), focusing on socioenvironmental influences, Successful Aging Theory (SAT), and key concepts from Table 2. We selected these three frameworks for several reasons. First, we selected AT because it is common in the literature on immigrant health. Moreover, as AT has been criticized in recent years for its focus on individual and cultural explanations [100, 101], we draw on Ward and Geeraet’s model as it includes ecological and Ecosocial dimensions. Only one study we reviewed on older adult immigrants’ health explicitly named SCT [102]. However, we selected SCT because, as demonstrated in Table 2, the key constructs in SCT were present in the literature, making SCT unstated but present. Specifically, the SCT socioenvironmental influences of social support, normative beliefs, such as the benefits of traditional medicine, and barriers and opportunities, such as the role of public transport or ELP on health were present in the literature. Finally, the concept of successful aging, was present in several studies we reviewed. SAT is a useful concept to incorporate because it focuses on the process of aging.

### Acculturation theory

Broadly defined, acculturation is the process by which one changes their behavior, values, and even primary language used, as they are immersed in another culture [83, 84, 99, 103]. In the literature, acculturation was applied as a definition, model, or framework. According to Berry, the most cited author on acculturation in the studies we reviewed, acculturation is bidimensional, meaning it involves negotiation with the new culture, and re-negotiation with the culture of origin [79, 81]. However, as we noted in the literature review section, there is a lack of clear and consistent definition of acculturation and application as a framework. Hence, we refer to Acculturation Theory (AT) as it denotes a multidirectional process of integration, negotiation, and potentially biculturalism [84, 99, 100], and we adapted, Ward’s and Geeraet’s [99] process model of acculturation. While Ward and Geeraet’s model is overall a comprehensive model, it has several limitations, which we address in the adapted model. First, the model is not specific to older adult immigrants, who are affected by aging-related challenges. Second, in our model, we place greater emphasis on one’s immigration history as a distinct phenomenon. The specific circumstances of one’s acculturation process, such as immigration history, should be considered [104]. Third, the model does not depict the manners that acculturative stressors may impact behaviors and influence health barriers and facilitators.

### Social cognitive theory

Social Cognitive Theory (SCT) has three major constructs: cognitive influences, socioenvironmental influences, and behavioral factors [105, 106]. We focus on socioenvironmental influences including observational learning, normative beliefs, barriers, opportunities, and social support [105, 106]. Socioenvironmental influences include not only how the physical environment promotes or hinders health, but also how the social environment, such as the social acceptability of behaviors as influenced by culture, which is especially pertinent for older adult immigrants, impacts health [105, 106]. AT relates to socioenvironmental influences as one’s pre-immigration and immigration experiences shape their socioenvironmental influences. While SCT is also a comprehensive theory, it has several limitations, which we seek to address in the adapted model. Specifically, while SCT considers socioenvironmental factors, it considers how those factors impact an individual’s behaviors [105].

### Successful aging theory

Successful Aging Theory (SAT) emphasizes three tenets of well-being, “low risk of disease and disease-related disability; maintenance of high mental and physical function; and continued engagement with life” [107]. A key tenet underlying SAT is that aging is a process [108]. Moreover, the four assumptions underlying SAT are: (1) Aging is a process of adaptation that gradually becomes more complicated, (2) the success or lack of success in aging depends on the complexity of adaptation required, for older adult immigrants this requires considerably more adaptation, (3) an individual’s choices impact their aging, and (4) aging changes people’s beliefs and values [108]. Annele et al. [109], operationalized SAT as a multifaceted concept [109]. Applied to older adult immigrants, SAT is age-specific to older adults, underscoring age-related physical and psychological challenges. Annele et al.’s model is modified from Fernandez-Ballesteros model [110]. While Annelle et al.’s model is comprehensive, it has several limitations which we seek to address in our adapted model. First, it focuses solely on the individual. Second, there is not a relationship between psychosocial factors and biomedical factors, even though these factors influence one another [111]. Finally, the very title of the model, ‘successful aging’, implies that there is a way to unsuccessfully age. Katz and Calasanti note that when older adults are asked about successful aging, “we learn that disability and disease are not necessarily experienced in terms of unsuccessful aging nor is successful aging a precondition of aging well” [112]. Hence, while activity, health, and productivity can all be good things for aging older adults, they do not dictate whether one self-assesses themselves as successfully aging. Therefore, in our adapted model, we focus on how multi-level factors interact to impact health, rather than an individual self-perception of aging success.

### A unified model

In Figure two, we illustrate our unified conceptual model. In addition to combining AT, SCT, and SAT, and addressing the aforementioned limitations, we added several aspects from Table 2. We incorporated elements from Table 2 to enhance the immigrant-specific elements of this model, and link elements from the literature as there was not a common model used. The unified model is called the Older Adult Immigrant Adapted Model for Health Promotion (OAHM).

A major benefit of this model, is that it includes concepts of aging, acculturation, immigration, and health, situated within an ecological context. From a public health perspective, this model illustrates how different dimensions interact specific to older adult immigrants, and spheres where prevention and intervention efforts could be targeted.

From a theoretical standpoint, the use of a clear framework, such as OAHM, would enhance studies [47]. We should note that we have used the terms model, framework, and theory rather interchangeably due to the number of different frameworks, models, theories, and uses of those terms, by authors in the literature. We recognize that these terms do have different definitions [113]. Thus, the OAHM is best considered a conceptual model. Additionally, when we use the phrase, ‘from a theoretical standpoint,’ we draw on Jaccard and Jacoby [114], to mean from the standpoint of recognizing the importance of relationships between concepts [114]. In essence, we argue that studies are better grounded, more clearly defined, and aid in a clearer understanding of outcomes when there is a ‘road-map’ of the relationships between concepts.

The three headings on the top-right, individual, interpersonal, and organizational and community, have double-headed arrows because they influence one another. For example, observational learning and normative beliefs impact service use. If service utilization is not acceptable due to observational learning and normative beliefs, then those important services may go unused. Lack of use of preventative care based on observational learning and normative beliefs was found to negatively impact the health of older adult immigrants in several studies [18, 94]. Individual, interpersonal, and community and organizational factors are directly linked to barriers and opportunities. For instance, a lack of a safe area to walk in or a lack of public transport may limit access to social support as well as contribute to poor physical function while enhanced ELP may provide opportunities for larger social networks and greater access to resources. At the same time, just as individual, interpersonal, and community and organizational factors interact with barriers and opportunities, so does cultural distance.

The interaction between one’s heritage culture and US culture, or intercultural contact, can be stress-inducing and requires negotiation and shifts in economic circumstances, languages, cultures, and roles, and influences barriers and opportunities. For example, for older adult immigrants, lack of SES resources due to migration circumstances may be a barrier to seeking adequate medical services. Additionally, while enhanced ELP may facilitate greater social support, both acquiring ELP and maintaining one’s native language are important for the health and well-being of older adult immigrants [61, 69, 90, 115, 116]. This cultural distance and negotiation influences the amount of adaptation required. Immigrants who migrated at an older age [117, 118] may face additional cultural and linguistic adaptation challenges. The amount of adaptation required, particularly if it is perceived as difficult adaptation, affects health aging, which affects overall health.

**Fig. 2:**
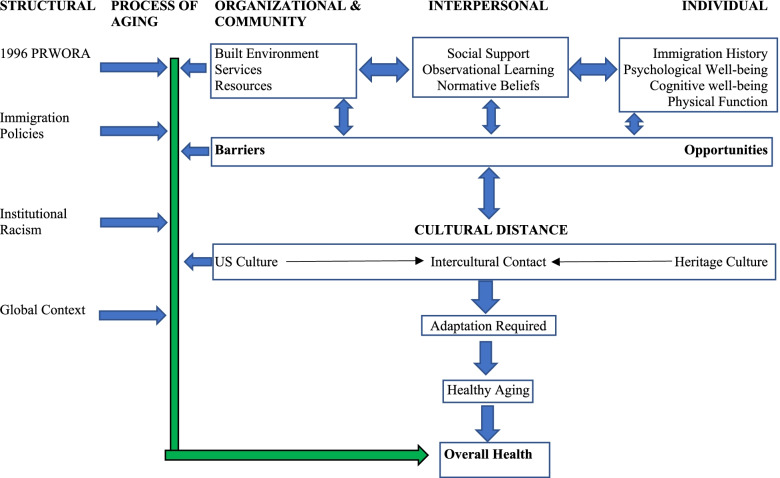
The Older Adult Immigrant Adapted Model for Health Promotion (OAHM)

The last two major sections in Fig. 2 are the structural and process of aging categories on the upper left. The reason arrows from the other sections point towards the process of aging section is because aging is a process, that gradually becomes more complicated [108], and it is an interaction between multiple factors that shape health. Moreover, in the process of aging, there is the aspect of time. Older adult immigrants continue to age which gradually creates more challenges. For example, feelings of isolation and depression may put an older adult immigrant at risk for worsened cardiovascular health [111]. This isolation may also act as a barrier to accessing health services, which negatively impact healthy aging and one’s overall health. Hence, from a public health perspective, promoting healthy aging and overall health requires interventional adaptation as one ages.

Finally, the structural section includes the 1996 PRWORA, immigration policies, institutional racism, and the global context. For example, the restrictions put in place by the 1996 PRWORA restrict a vulnerable population from receiving vital resources. Among older adult immigrants who immigrated after 1996, “Medicaid coverage significantly declined among older noncitizens but increased among older naturalized citizens after Welfare Reform” [119]. This lack of insurance, and also under-insurance among older adult immigrants generally [120], is a barrier and leads to a lack of usage of preventative care and costly emergency-room visits later [119]. Furthermore, as an example of how immigration policies impact health, the 1990 Immigration Act, “increased the refugee ceiling from 43,000 to 50,000 per year for peoples from the former Soviet Union” [94]. This, in combination with policy shifts in the FSU, allowed more refugees, specifically Jewish refugees, to leave the FSU. However, these policy shifts occurred decades after WWII, the holocaust, and during continued anti-Semitism in the FSU [121]. Thus, refugees from the FSU tended to be older and were exposed to negative conditions for a longer period of time before immigrating. Exposure to conditions, which Gubernskaya posits, is linked to worse health outcomes [18]. These policies influence the process of aging for older adult immigrants, and from a public health perspective when assessing health-influencing factors, the role of policy at certain points in time and in certain contexts, needs to be included. The OAHM illustrates how different factors interact to influence the health of older adult immigrants.

### Recommendations for future research

We have several recommendations for future research based on the literature we reviewed, and in the interest of coherence and concision, we provide three main recommendations for future research: Greater emphasis on context, a clear theoretical framework, and more robust and clearly defined measurement of concepts such as acculturation.

#### First recommendation

Future research with older adults should clearly explicate the context. Contextual information was missing from many of the studies we reviewed. Many studies we reviewed focused the background section of their study on a health outcome of interest without emphasizing the broader contextual factors influencing that outcome. Regardless of the type of study (qualitative, qualitative, mixed methods), context is crucial in aiding the researcher, readers, and wider audience in understanding the factors impacting that group. Context is crucial because factors affecting the health of older immigrants do not exist in isolation. These factors include history, policy, built environment, language, and institutional racism. To understand the barriers and facilitators of health among a particular immigrant group context is invaluable to illustrating that health does not exist in a vacuum. If we as public health professionals want to truly commit to increasing health equity, we need to understand the various manners in which different factors influence risk differently among specific older adult immigrant groups. For example, older adult immigrants from the FSU may seldom face discrimination based on race but face added barriers due to language; while older adult Latinx immigrants from Belize (an English-speaking country) may not face as significant language concerns, they may encounter institutional racism in the US. In essence, future studies should emphasize context.

#### Second recommendation

Secondly, we recommend that future studies apply a theoretical framework. Out of the 145 studies we reviewed, only 47 explicitly articulated that they used a theoretical framework. Using a clear theoretical framework enhances research [122]. Theory aids researchers in understanding the potential pathways and mechanisms potentially impacting the outcomes of interest in a study [47, 122]. As noted by Brazil et al., atheoretical approaches to research can lead to a “simple input/output” study [122, 123]. Hence, theory is important to shaping a comprehensive study; it informs the pathways impacting the variables of interest, which in turn may impact the questions asked in the study, study design, and evaluation tools. Future research, regardless of study type, should incorporate theory.

#### Third recommendation

Our third recommendation contains two steps. Firstly, most of the literature on older adult immigrants focused on those from South Korea, China, and the FSU. There was little literature on the health of older adult Latinx immigrants or those from the Middle East, and even less on older adult immigrants from the continent of Africa, or Black older adult immigrants from Latin America. Future research with older adult immigrants should pay greater attention to these groups. Secondly, as noted in the results section, acculturation was inconsistently defined and measured, leading to difficultly in determining to what extent one was able to conclude how the acculturative process impacted the health of older adult immigrants in the US. For example, common measures, such as citizenship status, ELP, and length of stay in the US are proxy measures and are confounded by other factors such as SES [124]. Hence, future studies in which the researcher is collecting primary data, should use robust measures of acculturation to truly attempt to capture the phenomenon they are attempting to capture. Such as Mao et al.’s examination of Behavioral Acculturation, Cognitive Acculturation, and Identificational Acculturation [60].

### Limitations

The main limitation of this review is that it is very broad. We did not focus on a particular health topic or population but instead the barriers and facilitators of older adult immigrant health in the US as a whole. However, for future studies or reviews, greater specificity is warranted. Moreover, we did not examine state-by-state differences regarding the differential application of the 1996 PWRORA. It is quite possible that, due to the restrictiveness of our search terms, important themes were not reflected in the literature we reviewed. For example, violence and pre-migration and migration experiences are not comprehensively discussed in this review, likely due to our restriction in search terms. Finally, this review does not address provider interactions with older adult immigrants. The most common recommendation in the articles we reviewed was that providers and staff who work with older adult immigrants should be better prepared to work with them. This recommendation, focused on interpersonal relationships, often lacked any explicit direction on how to integrate it. Only two articles engaged with staff, and Eckemoff found that hospice staff did not feel prepared or equipped to work with the older adult immigrants they were serving [125]. Future reviews should take this into account.

## Conclusion

Older adult immigrants in the United States are a large group, and unique factors inhibit or facilitate their health and well-being. As the population of immigrants, and older adult immigrants continues to grow in the US, the US medical and public health professionals need to be prepared to work with this large group. Hence, in this integrative review, we conducted a systematic review of 20 years of literature. We found that social support, isolation, mental health, activity participation, health insurance, and service use were the most commonly discussed barriers and facilitators of health. Moreover, a guiding framework was only used in one-third of the studies, and nearly every framework differed. Following our review of the literature, we examined AT, SAT, and SCT, and combined those theories with elements from the findings in the literature, to create the Older Adult Immigrant Adapted Model for Health Promotion (OAHM). Then, we explicated recommendations for future research, such as further illuminating acculturation, examining age-at migration, incorporating theory, understanding context, and conducting more research with Latinx older adult immigrants, and older adult immigrants from the continent of Africa. Public health strives to promote health and prevent adverse health outcomes. This is the first integrative review on the health of older adult immigrants in the US, and we strove to elucidate research recommendations, from a public health perspective, to promote the health of older adult immigrants in the US.

## Data Availability

Not applicable.
